# Conceptions about the mind-body problem and their relations to afterlife beliefs, paranormal beliefs, religiosity, and ontological confusions

**DOI:** 10.2478/v10053-008-0138-5

**Published:** 2013-09-20

**Authors:** Tapani Riekki, Marjaana Lindeman, Jari Lipsanen

**Affiliations:** Division of Cognitive Psychology and Neuropsychology, Institute of Behavioural Sciences, University of Helsinki, Finland

**Keywords:** dualism, mind-body problem, ontological confusions, religiosity, paranormal beliefs

## Abstract

We examined lay people’s conceptions about the relationship between mind and body and their correlates. In Study 1, a web survey (*N* = 850) of reflective dualistic, emergentistic, and monistic perceptions of the mind-body relationship, afterlife beliefs (i.e., common sense dualism), religiosity, paranormal beliefs, and ontological confusions about physical, biological, and psychological phenomena was conducted. In Study 2 (*N* = 73), we examined implicit ontological confusions and their relations to afterlife beliefs, paranormal beliefs, and religiosity. Correlation and regression analyses showed that reflective dualism, afterlife beliefs, paranormal beliefs, and religiosity were strongly and positively related and that reflective dualism and afterlife beliefs mediated the relationship between ontological confusions and religious and paranormal beliefs. The results elucidate the contention that dualism is a manifestation of universal cognitive processes related to intuitions about physical, biological, and psychological phenomena by showing that especially individuals who confuse the distinctive attributes of these phenomena tend to set the mind apart from the body.

## Introduction

One of the most long-standing scientific problems is the mind-body problem: How are the mind and the body (in particular, the brain) related to each other? Is the mind only temporarily attached to the body or is it possible that biological processes give rise to conscious thought and the feeling of free will? Throughout the centuries, scientists have offered a myriad of answers to the problem, and a host of current scientific theories have rooted the mind in the body, that is, in the brain and its processes. However, lay people’s conceptions of the mind-body relationship, as well as their associations, are not well known.

Scientific views on the mind-body relationship can be categorized and labeled in several ways (Kim, 2005). Studying lay people’s conceptions about the mind-body problem is challenging, as the conceptions and possible interaction between mind and body are diverse (Fahrenberg & Cheetham, 2000). In this study, we focus on three broad views and analyze whether lay people, who are not specialized in mind-body problem, see the human mind and body fundamentally the same, as physically independent (e.g., the mind can exist without a brain), or qualitatively different but interconnected. Here, we refer to these three positions of the mind-body relationships as monism, dualism, and emergentism. The three categories do not correspond to precise scientific theories.

The mind-body problem has been a central question in science. Although the debate continues and different perspectives on the mind-body problem are defended (e.g., Chalmers, 1996; Shear, 1999; Velmans & Schneider, 2007), since the 19th century - especially due to the rise of neurosciences - strong dualistic theories that emphasize mind and body as different substances and as entirely independent have mostly been rejected (Churchland, 1984; Dennett, 1993).

However, the way lay people think of the mind-body relation is poorly understood because only a few studies have addressed this issue and most have only analyzed dualism (e.g., Thalbourne, 1996). Demertzi et al. (2009) found that a majority of undergraduates and over one-third of healthcare workers considered the mind and brain to be separate, implying a dualistic view. In Stanovich’s (1989) study, 44% of psychology students accepted that thought processes cannot be brain processes, leading to the conclusion that there is a discrepancy between popular and scientific views of the mind. In addition, Fahrenberg and Cheetham (2000) found that (apart from psychophysically neutral preconceptions that did not take a stance on the relation of mind and body) dualistic conceptions were far more popular than monistic or emergentist conceptions among students from different disciplines.

Dualistic views can appear in several ways. They can be reflective beliefs (examined in the above studies) in that they are based on conscious pondering about mental phenomena and their relations to the brain, to substance, and to physical processes. Dualistic views can also be manifested as everyday beliefs that the mind can survive death, herein referred to as *common sense dualism*. Common sense dualism is widespread. For example, 73.3% of Americans and 43.2% of Europeans believe that the afterlife is possible (World Values Survey, 1991-2004). Preliminary evidence shows that common sense dualism is related to reflective dualism (Demertzi et al., 2009; Thalbourne, 1996). On this basis, we hypothesize that afterlife beliefs are positively associated with reflective dualism and negatively with the monistic view of the mind-body relationship (Hypothesis 1).

Several researchers have argued that common sense dualism is natural. Bering and Bjorklund (2004) propose that a natural disposition toward afterlife beliefs is a cognitive default, related to intuitions about biological, physical, and psychological phenomena. Similarly, other authors (Evans, 2008; Gjersoe & Hood, 2006) suggest that afterlife beliefs are a natural extension of how people think about human minds. Bloom (2004), for example, argues that our normal cognitive development in intuitive psychology and physics leads us to make sense of physical and mental entities differently, resulting in the assumption that bodies and souls are separate and independent. If dualism is a by-product of standard ontogenetic cognitive development, it makes religious ideas, such as gods and immortal souls, readily comprehensible (Bloom, 2007). Thus, we hypothesize that common sense and reflective dualism are associated with religiosity (Hypothesis 2).

Reflective and common sense dualism have also been shown to be positively related to non-religious paranormal beliefs, such as beliefs in incorporeal spirits (e.g., ghosts, out-of-body experiences), telepathy, psychokinesis, or faith healing (Stanovich, 1989; Thalbourne, 1996). Therefore, we hypothesize that dualism is also associated with paranormal beliefs (Hypothesis 3). As regards beliefs in incorporeal spirits, their relationship to dualism is understandable. However, no explanations have been offered as to why dualism would be associated with paranormal beliefs that have nothing to do with mind-body relations, such as belief in psychokinesis or faith healing (Stanovich, 1989; Thalbourne, 1996).

In this study, we test the possibility that making ontological confusions is a common factor linking these various forms of beliefs. What sets dualism apart from monistic and emergentistic views is that mental phenomena are believed to possess properties they cannot in reality have, such as independent existence. Similarly, religious and non-religious paranormal beliefs that do not cover mind-body relations are associated with confusions of the core attributes of physical, biological, and psychological phenomena: To put it in the terms of cognitive science of religion, folk theories contradict scientific theories about these attributes (Atran & Norenzayan, 2004; Barrett, 2000; Boyer, 2001).

By *core attributes*, we mean the fundamental attributes of evolutionarily important phenomena that children learn easily and universally at roughly the same age (Hirschfeld & Gelman, 1994; Spelke & Kinzler, 2007; Wellman & Gelman, 1998). For example, if the core properties of physical phenomena (especially independent existence and force) and biological organisms (e.g., living) are attributed to a human mind, it is easy to believe in souls that may live after the body has died. Similarly, assuming that such mental phenomena as thoughts or symbols can exert mechanical causal force on the external world, as physical entities do, enables beliefs in psychokinesis and astrology (Lindeman & Svedholm, 2012). Thus, what associates dualistic beliefs with all kinds of paranormal beliefs and makes understandable that these beliefs cluster together, is that they all confuse core attributes of psychological, biological, and physical phenomena. On this basis, we hypothesize that common sense dualism, reflective dualism, paranormal beliefs, and religiosity are related to ontological confusions about the core properties of psychological, physical, and biological phenomena (Hypothesis 4). If dualism and other paranormal beliefs are related to ontological confusions, it is possible that dualism is not the quintessential explanation for all religious and paranormal beliefs because there is a common denominator, ontological confusions, that theoretically explains why all these beliefs are associated. However, dualism might be understood as an essential building block for the more multifaceted and culture-specific forms of religiosity and paranormal beliefs but not as their quintessential explanation. Therefore, we test whether reflective and common sense dualism mediate the relationship between ontological confusions and religiosity and paranormal beliefs (Hypothesis 5).

## Study 1

### Method

#### Participants and procedure

Altogether 850 Finnish volunteers (59% women, 41% men, *M*_age_ = 30 years, range 16-66, *SD* = 11) participated in the study. Of the participants, 34.6% were university students and 7.3% were other students. The most common fields of university study were psychology (10.1%) and mathematics (6%). The rest of the university students studied 20 different subjects; one philosophy student was included. Of the participants who were not students, 32.5% were currently working in 95 different occupations, 12.9% were otherwise occupied, and 12.7% did not specify occupational status.

The highest completed educational degrees among the participants were basic education (17.7%), upper secondary level (38.1%), higher education (32.9%), and not specified (11.3%). Religious affiliations were the Evangelical Lutheran Church (46.2%), some other church (3.2%), none (38.4%), or no answer (12.2%). The participants were recruited via students’ mailing lists and several open internet message boards. No exclusion criteria for participation were applied. Confidentiality and voluntary participation were emphasized, and the study was performed in accordance with the ethical standards of the American Psychological Association (for these kinds of surveys, ethical approval is not needed in Finland). As compensation, all participants received a personal value profile based on the Portrait Values Questionnaire (PVQ scale; Schwartz et al., 2001) included in the survey. All questionnaires were in Finnish, and response times were not restricted.

#### Measures

##### Mind-body relationship

The scale was a modification of Stanovich’s (1989) 27-item Dualism scale. Because Stanovich’s scale addresses only dualism and many of its items are ambiguous, some items were excluded or simplified, and items concerning monism and emergentism were added. The new scale included 25 five-point items (1 = *strongly disagree*, 5 = *strongly agree*). A factor analysis with Varimax rotation identified three factors (see Appendix A for items and factor loadings). Variables, based on the factor loadings and scores, were termed *reflective dualism* (mind and body are qualitatively distinct), *emergentism* (mind and brains are qualitatively different but interdependent), and *monism* (mind and body are the same or fundamentally united), with reliability estimates (*rho*, Tarkkonen & Vehkalahti, 2005) of .87, .82, and .75, respectively.

##### Afterlife beliefs

Beliefs concerning biological and psychological processes that may continue after death were assessed with questions modified after Bering and Bjorklund (2004, Experiment 3). The questions were presented in a dichotomous form, for instance, “When a person is dead, is she or he still able to X” (“yes”, “no”). The scale included 22 items on biological processes (e.g., eating), psychobiological processes (e.g., being sleepy), perceptual processes (e.g., hearing), desire (e.g., wishes), emotions (e.g., feeling sad), and epistemic processes (e.g., thinking). The variable afterlife belief was an average score of the items (Cronbach’s ɑ = .94).

##### Paranormal beliefs

Paranormal beliefs (ɑ = .94) were measured with 22 items from the 26-item Revised Paranormal Belief Scale (Tobacyk, 2004). The 4-item subscale of Traditional Religiosity was excluded.

##### Religiosity

Sixteen items from the Fetzer Brief Multidimensional Measure of Religiousness/Spirituality (Neff, 2006) were used (ɑ = .97). We excluded five items (e.g., “I feel a deep sense of responsibility to reduce pain and suffering in the world”) from the 20 original items because even atheists could agree with them.

##### Ontological confusions

Ontological confusions were measured with 30 statements taken from the Core Knowledge Confusions scale (Aarnio & Lindeman, 2007; Lindeman et al., 2008). The participants were first presented with six clearly literal (e.g., “Sibelius was a composer”) or metaphorical practice sentences (e.g., “A surprising piece of news is a bombshell”) to describe the difference between metaphorical and literal sentences. After the examples, the participants were asked whether the statements were metaphorically or literally true. The originally 5-point scale was here used as a dichotomous scale (1 = *only metaphorically true*, 2 = *literally true*). The scale consisted of statements such as “Stars live in the sky” and “Force can sense a human being.” An average score of all items was calculated for ontological confusions (ɑ = .88). To disguise the purpose of the study, the scale also included four metaphorical and four literal statements presented randomly together with the 30 core know-ledge confusions statements. All participants (*n* = 18) who considered all four literal sentences as not literally true or all four metaphorical sentences as literally true were excluded.

### Results

Emergentism, *M* = 3.34, was more common than monism, *M* = 3.09, *t*(761) = 3.74, *p* < .001, and reflective dualism, *M* = 2.61, *t*(762) = -22.68,*p* < .001. The difference between monistic and reflective dualism was also significant, *t*(761) = -8.47, *p* < .001. The results remained significant after Bonferroni adjustments. A correlation analysis shows that Hypotheses 1-4 received support (Table 1). Reflective dualism was positively associated with afterlife beliefs, whereas monism was negatively associated with reflective dualism, religiosity, ontological confusions, afterlife beliefs, and paranormal phenomena. In addition, increased ontological confusions were associated with increased afterlife beliefs, reflective dualism, religiosity, and paranormal beliefs.

**Figure 1.**
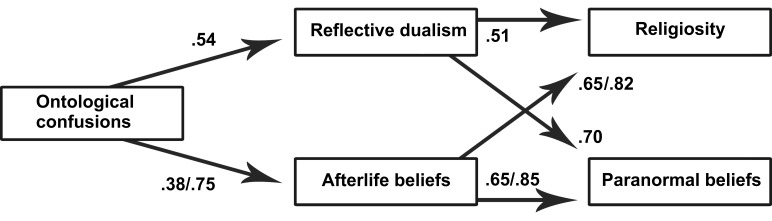
Path diagram of the relationship between ontological confusions, dualistic thinking, afterlife beliefs, religiosity, and paranormal beliefs (Study 1/Study 2)

Multiple regression analysis was conducted to test whether afterlife beliefs and reflective dualism mediate the relationship between ontological confusions and religiosity and paranormal beliefs (Figure 1, Table 2). A mediational hypothesis was tested following the instructions of Baron and Kenny (1986). A complete mediating effect was not found, and the hypothesis of a partial mediating effect was examined next using Sobel’s (1982) test. The partial mediation effect was significant (*p* < .001) in all analyses: ontological confusion - reflective dualism - religiosity, *Z* = 10.78; ontological confusion - afterlife beliefs - religiosity, *Z* = 8.97; ontological confusion - reflective dualism - paranormal beliefs, *Z* = 12.92; and ontological confusion - afterlife beliefs - paranormal beliefs, *Z* = 8.95. These results support Hypothesis 5that afterlife beliefs and reflective dualism mediate the effect between ontological confusions and paranormal beliefs and religiosity.

Core knowledge about mental, physical, and biological phenomena is claimed to stem from automatic implicit learning (Hirschfeld & Gelman, 1994; Spelke & Kinzler, 2007; Wellman & Gelman, 1998). Because we measured ontological confusions with self-reports, it is possible that the results reflect only participants’ explicit reasoning which might be different from their implicit beliefs. In Study 2, we tested the hypothesis that afterlife beliefs and reflective dualism mediate the relationship between ontological confusions and paranormal beliefs and religiosity with implicit measures of ontological confusions.

## Study 2

### Method

#### Participants

Seventy-four individuals (41 women, 33 men, *M*_age_ = 32 years, range 20-53), recruited through various mailing lists, internet message boards, and notice boards in esoteric bookstores and the Finnish Association of Skeptics, participated. Of the participants, 21 were university students and 53 were working in 34 different occupations. Recruitment was done with two announcements emphasizing opposite ends of the paranormal belief-skepticism continuum in the following way:

We are interested in people who relate to paranormal phenomena positively and/or consider them to be potentially true and/or who believe in an invisible spiritual world and … who consider paranormal phenomena to be against the laws of nature and thus impossible and/or who believe that there is no other reality than what we can perceive with our five senses.

#### Procedure and measures

Scales of ontological confusions, afterlife beliefs, and paranormal beliefs were the same as in Study 1. However, in contrast to Study 1, where participants assessed whether the ontological confusions were metaphorically or literally true, in Study 2 the participants judged if the statements were or were not literally true. This response format was used here because identifying ontological confusions as literally true is the crux of the matter. Moreover, forced-choice formats, in which respondents must choose between alternatives that represent different constructs, are limited in that they tend to reflect only the relative intraindividual strength of the assessed constructs and do not necessarily provide interindividual information (Clark & Watson, 1995).

Religiosity (ɑ = .86) was measured with the Traditional Religiosity subscale of the Revised Paranormal Belief Scale (Tobacyk, 2004). Participants first answered the afterlife beliefs and ontological confusions scales in a computerized version in a laboratory setting and then a pen and paper version of the paranormal beliefs scale.

Implicit ontological confusions were examined in a speeded condition, which is considered to eliminate the possibility to engage in effortful processes and to instead produce early developing default responses that are otherwise inhibited by later acquired knowledge (Bargh, 1989; Kelemen & Rosset, 2009; Wilkowski & Robinson, 2007). Ontological confusion statements were presented in the middle of a computer screen. Participants were asked to indicate their answer with a joystick. The true - false answering scale was located in the right corner of the screen. When the participants moved the joystick, a cursor on the scale moved accordingly. The participants were instructed to move the joystick as fast as possible and to press the button on the joystick when the cursor indicated their answer. All items were presented twice in counterbalanced order with a 4-s response time, falling thus between fast speeded and moderately speeded conditions (Kelemen & Rosset, 2009). In half of the items, true answers were given by pushing the joystick away from oneself, and false answers by pulling the joystick towards oneself. In the other half of the items, the answering was reversed. Answers given without pushing or pulling the joystick to either of the ends were coded as misses. A joystick method was used because we originally planned to combine the approach-avoidance method (Chen & Bargh, 1999) with judgments made in the speeded condition. However, as the joystick did not work similarly to the levers that are typically used in the approach-avoidance method, only results concerning speeded judgments are reported. Six participants were excluded from analysis because they had answered that all metaphoric or literal sentences were literally true or not literally true, respectively. The implicit ontological confusion variable was formed from the average answer (true - false) to the scale. Afterlife beliefs were measured with the same procedure as ontological confusions, and the average score was used to form the afterlife beliefs variable.

### Results

There was a significant indirect effect of implicit ontological confusions via afterlife beliefs to paranormal beliefs (*b* = 1.57, bias corrected *CI* [0.88, 2.20] and to religiosity (*b* = 1.86, bias corrected *CI* [0.98, 2.71]).The direct effects of implicit ontological confusions to superstition and religiosity were statistically significant, showing a partial mediation effect of afterlife beliefs. The model explained 77.8% of the variance of paranormal beliefs, *F*(2, 69) = 120.88, *p* < .001, and 73.3% of the variance of religiosity, *F*(2, 69) = 94.79, *p* < .001. To compensate for the small sample size and the skewed distribution of paranormal beliefs and to obtain reliable estimates for mediation effects, the regression analysis was done with 1,500 bootstrapped resamples (Bollen & Stine, 1990; Shrout & Bolger, 2002). Analysis was done using an SPSS macro (Preacher & Hayes, 2008). The results, presented in Figure 1 and in Tables 1 and 2, lend further support for Hypothesis 5. It should be noted that the exceptionally high correlations in Study 2 are probably inflated because strong believers and confirmed skeptics were overrepresented in the sample.

**Table 1.** Correlations Between Study Variables

	1	2	3	4	5	6	7
1. Reflective dualism							
2. Emergentism	.06						
3. Monism	-.14**	-.11*					
4. Afterlife beliefs	.50**	.15*	-.32**		.82**	.85**	.75**
5. Religiosity	.51**	.18**	-.37**	.65**		.90**	.78**
6. Paranormal beliefs	.70**	.17**	-.33**	.65**	.71**		.80**
7. Ontological confusions	.54**	.06	-.14**	.38**	.39**	.58**	

*Note*. Below diagonal: Study 1. Above diagonal: Study 2. *p < .01. **p < .001.

**Table 2.** Relationships of Ontological Confusions With Religiosity and Paranormal Beliefs When the Mediating Effects of Dualistic Thinking and Afterlife Beliefs Are Assumed

Independent variable	Dependent variable	β	*t*	*R*^2^
Study 1				
Ontological confusions	Religiosity	.17	3,87**	.29
Reflective dualism		.43	9,92**	
Ontological confusions	Religiosity	.17	5,11**	.46
Afterlife beliefs		.59	17,49**	
Ontological confusions	Paranormal beliefs	.29	8,36**	.54
Reflective dualism		.53	15,38**	
Ontological confusions	Paranormal beliefs	.38	12,45**	.55
Afterlife beliefs		.51	16,62**	
Study 2				
Ontological confusions	Religiosity	.31	4,34**	.78
Afterlife beliefs		.27	6,71**	
Ontological confusions	Paranormal beliefs	.31	4,33**	.73
Afterlife beliefs		.27	10,99**	

Note**p < .001.

## Discussion

The results showed that the most popular view was that the mind and the body (e.g., the brain) are qualitatively different, but that the mind is nevertheless dependent on the body (i.e., emergentism). The least favored view was monism, which states that the mind and the brain are fundamentally and physically the same. Endorsement of reflective dualism fell between emergentism and monism. As in earlier studies (Demertzi et al., 2009; Thalbourne, 1996) and specifically in Stanovich’s (Stanovich, 1989) study, whose dualism scale we modified here, the mean of reflective dualism was near the theoretical mean. However, our finding that emergentism was the most preferred view was new and suggests that the participants’ views about the mind-body relationship were more in line with today’s scientific views than thus far assumed (Demertzi et al., 2009; Fahrenberg & Cheetham, 2000; Stanovich, 1989). It is important to note that about half of the participants were either university students or had university education and that the percentage of participants without a religious denomination was high (38.4%). These factors may have inflated non-dualistic views.

We also found that if the mind was seen as dualistically detached from the brain, more afterlife beliefs were endorsed. The results suggest that even though lay people may often not ponder reflectively about the mind-body problem (Stanovich, 1989), when they are asked to do so, the reflective views do not contradict their everyday beliefs. Afterlife beliefs emerge early in life and are already present in childhood (e.g., Bering & Bjorklund, 2004; Bloom, 2007), while the reflective views about the mind-body relationships develop later in life. The reason why some people rely more on emergentism or monism than on other views is unknown, but the results show that if this happens afterlife beliefs decrease as well.

Reflective dualism and afterlife beliefs were strongly related to religiosity, while emergentism was only slightly associated with religiosity and monism was negatively associated with religiosity. The links between reflective dualism and afterlife beliefs and religiosity are in line with the arguments that naturally and universally emerging dualism make religious arguments comprehensible and easy to adopt (Bering, 2006; Bloom, 2007). However, our findings broaden this view by drawing attention to individual differences in adulthood: People who retain the view that the mind and the brain are separate tend to be religious, while those who have emergentistic or monistic views are less religious.

Reflective and common sense dualism were not only connected to religiosity, but they were also strongly related to paranormal beliefs (such as belief in astrology, omens of bad luck, extrasensory perception, and psychokinesis). Similar findings for reflective dualism have been obtained earlier (Stanovich, 1989; Thalbourne, 1996), but the reasons why separating the mind from the body should co-vary with beliefs in, say, telepathy, have not been clarified.

Coupled with earlier studies (Lindeman & Aarnio, 2007; Lindeman et al., 2008), our results may offer a theoretically parsimonious explanation for the above findings. Reflective and common sense dualism as well as religiosity and paranormal beliefs were strongly and positively related to explicit (Study 1) and implicit (Study 2) ontological confusions about the fundamental properties of mental, biological, and physical phenomena. For example, the more lifeless entities (e.g., force, stars) were bestowed with life or intentions, and the more emotions were attributed with the properties of material objects (e.g., sadness literally *moves in the stomach*), the more dualism, paranormal beliefs, and religiosity were endorsed. Categories such as mental phenomena, animate and living organisms, lifeless material objects, and physical processes are all ontologically basic categories, and their distinct properties cannot be borrowed to characterize an entity in another ontological category without making a category mistake (Carey, 1985; Keil, 1979; Ryle, 1949). Based on these results, we suggest that reflective dualism, afterlife beliefs, and paranormal and religious beliefs are different facets of a more general tendency to extend the distinctive attributes of physical, biological, or psychological phenomena inappropriately to other domains. In addition, the mediation effects found in the two studies suggest that mind-body dualism may be an important step in understanding how religious and paranormal beliefs evolve. If the universal, naturally emerging idea that mental processes are different from physical processes progresses to a radical view of total independency of mental phenomena from the body, it may serve as fertile ground for various, culturally specific religious and paranormal beliefs.

This explanation does not contradict arguments that dualism is based on domain-specific cognitive systems, one for dealing with material (living or lifeless) objects, the other for psychological phenomena (Bering, 2006; Bering & Bjorklund, 2004; Bloom, 2004). Nonetheless, our findings suggest that it is not the ability to differentiate mental phenomena (e.g., thinking of a car) from physical phenomena (e.g., a car) alone that brings about dualism. After all, we can all make this distinction, but we are not all dualists or afterlife believers. Rather, it is the individual tendency to believe that mental phenomena are like independent material objects and that physical, inanimate phenomena can have mental properties that predispose to both reflective dualism and belief in the afterlife. In short, dualism may not stem directly from innate universal cognitive processes, but from a bias to which some people are more inclined than others.

The argument that confusions about the core properties of mental, biological, and physical phenomena may explain dualistic views about mind and body raises the question about the development of ontological knowledge because studies with children show that a similar type of confusions decreases at preschool age (Piaget, 1929/1951; Rakison & Poulin-Dubois, 2001; Rosengren, Johnson, & Harris, 2000). However, studies with adults show that when the capacity to inhibit intuitive reasoning is impaired, ontological confusions increase (Kelemen & Rosset, 2009; Svedholm & Lindeman, 2013). Kelemen, Rottman, and Seston (2012), for example, showed that when placed under cognitive-processing restrictions, even professional physical scientists explain biological processes in mental terms such as purpose and intentional designs (e.g., “Germs mutate in order to become drug resistant”). These findings suggest that while development of analytical thinking and cognitive inhibition as well as cultural input may suppress dualism,teleological explanations, and other cognitive defaults, they do not necessarily always replace them. In education, attention should be paid to possible ways to decrease ontological confusions, as these confusions may contribute to resistance to scientific information about, for instance, the mind and its functions (Bloom & Weisberg, 2007).

There are several limitations of the study. First, lay perceptions about mind-body relationships are not comparable with their scientific counterparts because the first are typically simpler and they can be unconscious and not verbalizable, whereas scientific views are complex, abstract, and detailed. Similarly, ontological confusions about physical, biological and mental phenomena are often implicit, and thus, difficult to access with self-report measures. Furthermore, and despite the high reliability of the scale, distinguishing literal and metaphorical meanings of the core knowledge statements may be difficult. Therefore, a different response was required in Study 2 than in Study 1, which may reduce the comparability of the results. However, we consider this unlikely because both response formats have been used in earlier studies (e.g., Lindeman, 2011; Lindeman & Aarnio, 2007; Lindeman et al., 2008; Svedholm & Lindeman, 2013) but no differences in scale reliabilities or correlates of ontological confusions have been observed. However, to validate the present conclusions, the way that study participants understand the idea of a metaphor, a literal statement, and a non-literal statement should be analyzed together with core knowledge confusions in future studies. Also, the present tests of mediation cannot establish causal links definitively, and therefore, experimental data are needed to validate the conclusion that belief in the afterlife and dualism are both different facets of ontological confusion. Nevertheless, statistically significant mediation effects provide evidence that for these variables this mediation pattern is more plausible than another (Baron & Kenny, 1986; Shrout & Bolger, 2002).

Mind-body conceptions may also have practical implications. It has been suggested that seeing mental illnesses as “all in the mind” may lead to ill-founded distinctions between mental and physical illnesses and may even influence judges’ perspectives when sentencing criminals (Kendell, 2001; see also Gray, Knickman, & Wegner, 2011). Similarly, Fahrenberg and Cheetham (2000, 2007) found that a majority of their participants (and dualists in particular) agreed that mind-body conceptions affect psychologists’ and doctors’ choice of diagnostic and treatment methods. These observations call for empirical studies where healthcare and other professionals’ mind-body conceptions are examined together with the decisions they make about other people, both in experimental and in natural settings. Until future studies address these issues, we may only speculate that a strong dualism can lead to unfounded judgments about people in everyday and professional life.

## Appendix A

Ten items described mind and body as qualitatively distinct and independent, assessing different forms of reflective dualism (Harre, 2001). Six items assessed emergentic thinking by describing brain and mind as qualitatively different yet interdependent (Chalmers, 2002). Monism was measured with nine items that highlighted the fundamental unity of mind and brain (Churchland, 1984).

**Table A1.** Loadings of the 26 Items in the Mind-Body Relationship Scale on Three Factors

Item	Factor 1	Factor 2	Factor 3
Factor 1: Reflective dualism			
Minds are in principle independent of bodies, to which they are only temporarily attached.^a^	.72	.09	-.23
The mind is a special form of energy, currently unknown to man, that is in contact with the brain and affects it.^a^	.72	.23	-.15
Thought processes cannot be just brain processes.^a^	.68	.24	-.38
The mind is immaterial and it works with the brain to generate our behavior.	.68	.38	-.19
The consciousness of myself does not die with my physical body.	.66	.06	-.28
The body belongs to the world of material and natural laws. The mind is a different kind of existence a spiritual way of being.	.65	.44	-.20
The body is material and the mind is immaterial.	.64	.38	-.09
Some mental processes have no connection to brain processes.^a^	.64	.13	-.19
The mind is not part of the brain, but it affects the brain.^a^	.60	.20	-.21
Mental states are activities of my nervous system.	-.58	-.10	-.43
The mind as a whole is made up of substance and material processes.	-.58	-.35	.47
The mind and the brain are totally different things.^a^	.57	.09	-.17
Factor 2: Emergentism			
The activity of the mind is based on the brain, but it is also something more than just the outcome of brain activity.	.29	.72	-.30
The mind is based on brain activity, but the mind as a whole is more than only the activity of the brain.	.24	.71	-.31
The operation of the mind is generated from the activity of the brain, but the operation of the mind is qualitatively different from the operation of the brain.	.02	.71	-.02
The mind is based on the brain, but the mind also has attributes that the chemical and physiological events of the brain do not have.	.33	.62	-.27
Although the mind is based on the brain, the mind is more than a biological process that takes place in the brain.	.44	.60	-.33
The mind is based on the activity of the brain, but one cannot perceive the attributes of the mind as such in the brain.	.08	.50	-.26
Factor 3: Monism			
When people talk about their minds, they are really just talking about what their brain is doing.^a^	-.42	-.32	.62
The mind is a physiological state of the brain.	-.51	-.26	.58
The word “mind” can be used as a shorthand term for the complicated things that my brain does.^a^	-.37	-.17	.58
Hundreds of years in the future, when we know how the brain states and thoughts are related, it might be possible for a physiologist to measure my brain states and know what I am thinking.^a^	-.11	-.30	.51
Not much would be lost if we dropped the word “mind” from our vocabularies. For example, rather than say “I made up my mind”, we might say “My brain decided” because the mind is the same as the brain.^a^	-.37	-.40	.46
For each thought that I have, there exists a certain state that my brain is.^a^	-.40	-.08	.44
When our knowledge about physiology increases, we may say “My c-fibers are sending nerve impulses”, instead of “I’m pain.”a	-.10	-.24	.41

^a^Item from the original Dualism scale (Stanovich, 1989).

## References

[R1] Aarnio K., Lindeman M. (2007). Religious people and para-normal believers: Alike or
different?. Journal of Individual Differences.

[R2] Atran S., Norenzayan A. (2004). Religion’s evolutionary landscape: Counterintuition,
commitment, compassion, communion.. Behavioral and Brain Sciences.

[R3] Bargh J. A., Uleman J. S., Bargh J. A. (1989). Conditional automaticity: Varieties of automatic influence in
social perception and cognition.. Unintended thought.

[R4] Baron R. M., Kenny D. A. (1986). The moderator-mediator variable distinction in social
psychological research: Conceptual, strategic, and statistical
considerations.. Journal of Personality and Social Psychology.

[R5] Barrett J. L. (2000). Exploring the natural foundations of religion.. Trends in Cognitive Sciences.

[R6] Bering J. M. (2006). The folk psychology of souls.. Behavioral and Brain Sciences.

[R7] Bering J. M., Bjorklund D. F. (2004). The natural emergence of afterlife reasoning as a developmental
regularity.. Developmental Psychology.

[R8] Bloom P. (2004). Descartes’ baby: How the science of child development explains
what makes us human..

[R9] Bloom P. (2007). Religion is natural.. Developmental Science.

[R9a] Bloom P., Weisberg D. S. (2007). Childhood origins of adult resistance to science.. Science.

[R10] Bollen K. A., Stine R. (1990). Direct and indirect effects: Classical and bootstrap estimates of
variability.. Sociological Methodology.

[R11] Boyer P. (2001). Religion explained..

[R12] Carey S. (1985). Conceptual change in childhood..

[R13] Chalmers D. J. (1996). The conscious mind: In search of a fundamental theory..

[R14] Chalmers D. J., Clayton P., Davies P. (2002). Strong and weak emergence.. The re-emergence of emergence.

[R15] Chen M., Bargh J. A. (1999). Consequences of automatic evaluation: Immediate behavioral
predispositions to approach or avoid the stimulus.. Personality and Social Psychology Bulletin.

[R16] Churchland P. M. (1984). Matter and consciousness..

[R17] Clark L. A., Watson D. (1995). Constructing validity: Basic issues in objective scale
development.. Psychological Assessment.

[R18] Demertzi A., Liew C., Ledoux D., Bruno M., Sharpe M., Laureys S. (2009). Dualism persists in the science of mind.. Annals of the New York Academy of Sciences.

[R19] Dennett D. C. (1993). Consciousness explained..

[R20] Evans J. S. B. T. (2008). Dual-processing accounts of reasoning, judgment, and social
cognition.. Annual Review of Psychology.

[R21] Fahrenberg J., Cheetham M. (2000). The mind-body problem as seen by students of different
disciplines.. Journal of Consciousness Studies.

[R22] Fahrenberg J., Cheetham M. (2007). Assumptions about human nature and the impact of philosophical
concepts on professional issues: A questionnaire-based study with 800
students from psychology, philosophy, and science.. Philosophy, Psychiatry, & Psychology.

[R23] Gjersoe N. L., Hood B. M. (2006). The supernatural guilt trip does not take us far
enough.. Behavioral and Brain Sciences.

[R24] Gray K., Knickman A. T., Wegner D. M. (2011). More dead than dead: Perceptions of persons in the persistent
vegetative state.. Cognition.

[R25] Harre R., Smelser N. J., Baltes P. B. (2001). Mind-body dualism.. International encyclopedia of the social & behavioral
sciences.

[R26] Hirschfeld L. A., Gelman S. A. (Eds.). (1994). Mapping the mind: Domain specificity in cognition and culture..

[R27] Keil F. C. (1979). Semantic and conceptual development: An ontological
perspective..

[R28] Kelemen D., Rosset E. (2009). The human function compunction: Teleological explanation in
adults.. Cognition.

[R29] Kelemen D., Rottman J., Seston R. (2012). Professional physical scientists display tenacious teleological
tendencies: Purpose-based reasoning as a cognitive default.. Journal of Experimental Psychology: General. Advance online
publication..

[R30] Kendell R. E. (2001). The distinction between mental and physical
illness.. The British Journal of Psychiatry.

[R31] Kim J. (2005). Philosophy of mind (enlarged and revised 2nd edition)..

[R32] Lindeman M. (2011). Biases in intuitive reasoning and belief in complementary and
alternative medicine.. Psychology and Health.

[R33] Lindeman M., Aarnio K. (2007). Superstitious, magical, and paranormal beliefs: An integrative
model.. Journal of Research in Personality.

[R34] Lindeman M., Cederstrom S., Simola P., Simula A., Ollikainen S., Riekki T. (2008). Sentences with core knowledge violations increase the size of
N400 among paranormal believers.. Cortex.

[R35] Lindeman M., Svedholm A. M. (2012). What’s in a term? Paranormal, superstitious, magical, and
supernatural beliefs by any other name would mean the same.. Review of General Psychology.

[R36] Neff J. A. (2006). Exploring the dimensionality of “religiosity” and
“spirituality” in the Fetzer multidimensional
measure.. Journal for the Scientific Study of Religion.

[R36a] Piaget J. (1929/1951). The child’s conception of the world..

[R37] Preacher K. J., Hayes A. F. (2008). Asymptotic and resampling strategies for assessing and comparing
indirect effects in multiple mediator models.. Behavior Research Methods.

[R38] Rakison D. H., Poulin-Dubois D. (2001). Developmental origin of the animate-inanimate
distinction.. Psychological Bulletin.

[R39] Rosengren K. S., Johnson C. N., Harris P. L. (Eds.). (2000). Imagining the impossible. Magical, scientific, and religious thinking in
children..

[R40] Ryle G. (1949). The concept of mind..

[R41] Schwartz S. H., Melech G., Lehmann A., Burgess S., Harris M., Owens V. (2001). Extending the cross-cultural validity of the theory of basic
human values with a different method of measurement.. Journal of Cross-Cultural Psychology.

[R42] Shear J. (Ed.). (1999). Explaining consciousness: The hard problem..

[R43] Shrout P. E., Bolger N. (2002). Mediation in experimental and nonexperimental studies: New
procedures and recommendations.. Psychological Methods.

[R44] Sobel M. E., Leinhart S. (1982). Asymptotic intervals for indirect effects in structural equations
models.. Sociological methodology.

[R45] Spelke E. S., Kinzler K. D. (2007). Core knowledge.. Developmental Science.

[R46] Stanovich K. E. (1989). Implicit philosophies of mind: The dualism scale and its relation
to religiosity and belief in extrasensory perception.. The Journal of Psychology.

[R47] Svedholm A. M., Lindeman M. (2013). The separate roles of the reflective mind and involuntary inhibitory
control in gate keeping paranormal beliefs and the underlying intuitive
confusions. British Journal of Psychology. Advance online
publication..

[R48] Tarkkonen L., Vehkalahti K. (2005). Measurement errors in multivariate measurement
scales.. Journal of Multivariate Analysis.

[R49] Thalbourne M. A. (1996). Belief in life after death: Psychological origins and
influences.. Personality and Individual Differences.

[R50] Tobacyk J. J. (2004). A revised paranormal belief scale.. The International Journal of Transpersonal Studies.

[R51] Velmans M., Schneider S. (Eds.). (2007). The Blackwell companion to consciousness..

[R52] Wellman H. M., Gelman S. A. (1998). Knowledge acquisition in foundational domains. In D. Kuhn & R. S.
Siegler (Eds.), Handbook of child psychology. Cognition, perception, and
language (2nd ed., pp. 523-573)..

[R53] Wilkowski B. M., Robinson M. D. (2007). Keeping one’s cool: Trait anger, hostile thoughts, and the
recruitment of limited capacity control.. Personality and Social Psychology Bulletin.

